# Evaluation of hip abductor and adductor strength in the elderly: a reliability study

**DOI:** 10.1186/s11556-017-0174-6

**Published:** 2017-04-24

**Authors:** Simone Gafner, Caroline H. G. Bastiaenen, Philippe Terrier, Ilona Punt, Serge Ferrari, Gabriel Gold, Rob de Bie, Lara Allet

**Affiliations:** 1 Department of Physiotherapy, HES-SO//University of Applied Sciences and Arts of Western Switzerland, Geneva, Switzerland; 2 Department of Community Medicine, University Hospitals and University of Geneva, Geneva, Switzerland; 3 Department of Epidemiology, Research program Functioning and Rehabilitation CAPHRI, Maastricht University, Maastricht, The Netherlands; 4 Department of Epidemiology, Maastricht University, Maastricht, The Netherlands; 5 Clinique romande de réadaptation SUVACare, Sion, Switzerland; 6 Institute for Research in Rehabilitation, Sion, Switzerland; 7 Department of Internal Medicine Specialties, University Hospitals and University of Geneva, Geneva, Switzerland; 8 Department of Rehabilitation and Geriatric University Hospitals and University of Geneva, Geneva, Switzerland

**Keywords:** Geriatrics, Elderly, Muscle strength, Hip, Test, Measurements, Reliability

## Abstract

**Background:**

In elderly individuals an increased muscle strength contributes to the diminution of the falls risk and associated adverse events. An increasing interest in lateral control exists due to the fatal consequences of postero-lateral falls. Therefore a proper assessment of frontal plane hip muscle strength in elderly is important but remains challenging.

Therefore we aimed to investigate the feasibility and repeatability of a hip abductor and adductor maximum voluntary isometric strength (MVIS) and rate of force generation (RFG) test in elderly. This represents an initial step in the development process of a new and clinically relevant test that could lead to more specific treatment protocols for this population.

**Methods:**

In this measurement focused study hip abduction (ABD) and adduction (ADD) MVIS and RFG were tested twice within one to three hours with a dynamometer fixed to a custom made frame in a geriatric population including fallers and non-fallers. Intraclass correlation coefficient (ICC_agreement_), standard error of measurement (SEM), and smallest detectable difference (SDD) were determined.

**Results:**

All recruited persons (*N* = 76; mean age (SD) 80.46 (7.05) years old) completed the tests. The average time needed to complete the strength tests was 10.58 min. (1.56) per muscle group. The reliability of the hip ABD and ADD was high with ICC’s_agreement_ ranging from 0.83 to 0.97. The SDD varied between 18.1 and 81.8% depending on the muscle group and type of strength that was evaluated.

**Conclusion:**

Hip abductor and adductor strength measures in older person are feasible and reliable. However, the significance of moderate changes in these measurements may be limited by the large SDD and SEM. Therefore, physical therapist should be careful when using this measure for assessing the progress of an individual person in a daily clinical use.

## Background

Approximately 50% of Swiss people older than 75 years live alone and most of them show a clinically relevant decline in their ability to execute daily life activities [1]. These disabilities are linked to decreased physiological capabilities, such as diminished muscle strength and power production. Reduction in muscle strength in the elderly increases the risk of mobility limitation [2–8], falls [9] and mortality [10–13].

Shumway-Cook et al. [14] showed that the annual health care costs were $2,000 (29%) higher in older adults reporting one fall during the previous year comparing to non-fallers and $5,600 (79%) higher among those reporting recurrent falls during the previous year. Therefore, the maintenance of muscle strength and power with advancing age is of great clinical importance as it contributes to diminish the risk of falls, fractures and associated adverse events, and hence, is implicated when trying to maintain independence [15, 16].

The role of lower limb muscle groups during walking patterns has been described by Perry [17]. Frontal plane hip muscles (abductors/adductors) play an important role during walking as these muscles are essential to control the head, arms and trunk during single leg stance phase. They further allow a proper swing foot placement after the swing phase [17, 18]. An increasing interest in lateral control is emerging, given that impairments in this domain are thought to increase falls risk [19, 20]. Recent findings in nonlinear gait analysis even suggest that gait stability in the frontal plane is of interest for predicting fall risk [21, 22]. Finally, lateral and postero-lateral falls have greater hip injury potential, including hip fractures, than falls in other directions [23–25].

Our previous work showed that frontal plane hip strength might be able to compensate for distal neuromuscular deficits in persons with distal symmetric neuropathy (DSP) during gait in challenging circumstances as it did during uni-pedal stance time (UST) [26]. Muscle strength (maximum voluntary isometric strength (MVIS)) was particularly important for static tasks like balancing on one leg, whereas the power (rate of force generation (RFG)) was important for safe ambulation [19, 26, 27]. The absence of therapy to restore nerve health in people with age-related declining peripheral nerve function makes it important to find innovative strategies which allow patients to compensate for these distal nerve function losses. The preservation of the ability to increase hip strength in these patients might enable them to compensate these distal sensory deficits through other bodily functions and hence, safely maintain mobility and prevent falls.

However, effective and correct assessment of frontal plane hip muscle strength and power in the elderly remains a challenge. The clinical experience show that an assessment with an isokinetic system like the Biodex is time consuming and it can be difficult to install older people on such isokinetic apparatus. In addition, only few rehabilitation centers have such equipment. Therefore daily clinical use of the biodex apparatus to measure hip strength in older persons, in particular hip abduction and adduction strength which necessities a side-lying or supine position, seems unrealistic. The fact that only a few studies exist [28, 29] that measure hip abduction and adduction strength in older persons at risk of falling underlines the difficulty of properly measuring hip frontal plane strength.

The aim of this study was to investigate the feasibility and repeatability of a newly developed hip abductor and adductor MVIS and RFG test with a dynamometer fixed to a custom made frame in a geriatric population including fallers and non-fallers. This is an important first step in the development process of this innovative measure for clinical practice as it could lead to the development of more specific treatment protocols for this population. To do so we recruited men and women ≥ 65 years of age, both with and without a history of falls, and assessed: 1) how many of the recruited persons could successfully finish the hip abductor and adductor MVIS and RFG test, 2) the time needed to administer the test, 3) the test-retest reliability, 4) the measurement error (SEM) as well as 5) the smallest detectable difference (SDD).

We hypothesized that the hip abductor and adductor MVIS and RFG test with a dynamometer fixed to a custom made frame is feasible and would reliably assess hip strength in a population consisting of both fallers and non-fallers with intraclass correlation coefficients (ICC’s_agreement_) of ≥ 0.7.

## Methods

### Subjects and recruitment

Overall 76 older persons were recruited from a geriatric hospital that is part of the Geneva University Hospitals and in an outpatients practice in Switzerland. Half of them (38 persons consisting in 19 fallers and 19 non-fallers) underwent the abduction and the other half of them (38 persons consisting in 19 fallers and 19 non-fallers) the adduction test.

To be included participants had to be over 65 years. They were excluded if their medical record contained a history or evidence of any significant central nervous system dysfunction (i.e., hemiparesis, myelopathy or cerebellar ataxia), any neuromuscular disorder other than a distal symmetric peripheral neuropathy (i.e., myopathy or myasthenia gravis) or evidence of vestibular dysfunction. In addition we excluded persons with a moderate or severe dementia (Minimal Mental State Exam (MMSE) < 18) which did not allow them to understand the study information and the necessary instructions, and persons with a severe sepsis, metastatic cancer, angina or angina-equivalent symptoms with exercise. We also excluded persons with a plantar skin sore or joint replacement within the previous year and persons with a non-consolidated fracture, significant musculoskeletal deformity (i.e., amputation, Charcot changes), lower limb or spinal arthritis or pain that limited proper execution of the test.

Within the recruited population a faller was defined as a person who had one or more falls during the last 12 months. Non-fallers were defined as participants who did not fall during the last 12 months. A fall was defined as an event which results in a person coming to rest inadvertently on the ground or floor or other lower level [30].

### Ethics

The study was approved by the ethical commission in Geneva (CCER - 14–235). All participants signed the written informed consent (declaration of Helsinki) after having received information about the study and time to decide about participation.

### Dynamometer

An analog dynamometer (SENSIX®, Poitiers, France) able to measure forces between 0 to 667 N with a precision of 0.002 N was used to measure hip abductor strength (N). The calibrated analog dynamometer was coupled with the DELSYS System (Trigno sensor, DELSYS, INC Boston; MA) that digitalized the analog output (3.3 V) with a sampling rate of 1926 Hz and a 16 bit resolution.

### Procedure

The force of hip abductors and adductors was measured in side-lying position by an experienced physical therapist. This position was previously described as the most valid and reliable position to measure hip abductor strength in young people [29]. In addition, the influence of hip abductor strength of the contra-lateral leg is reduced in the side-lying position compared to the supine or standing position [29]. The same examiner repeated the whole test procedure on the same day with a break of minimum one and maximum three hours in between. This time was estimated as being enough to fully recover, but not too long to have a change in the strength performance of the elderly frail persons.

### Hip Abduction Test

**Fig. 1:**
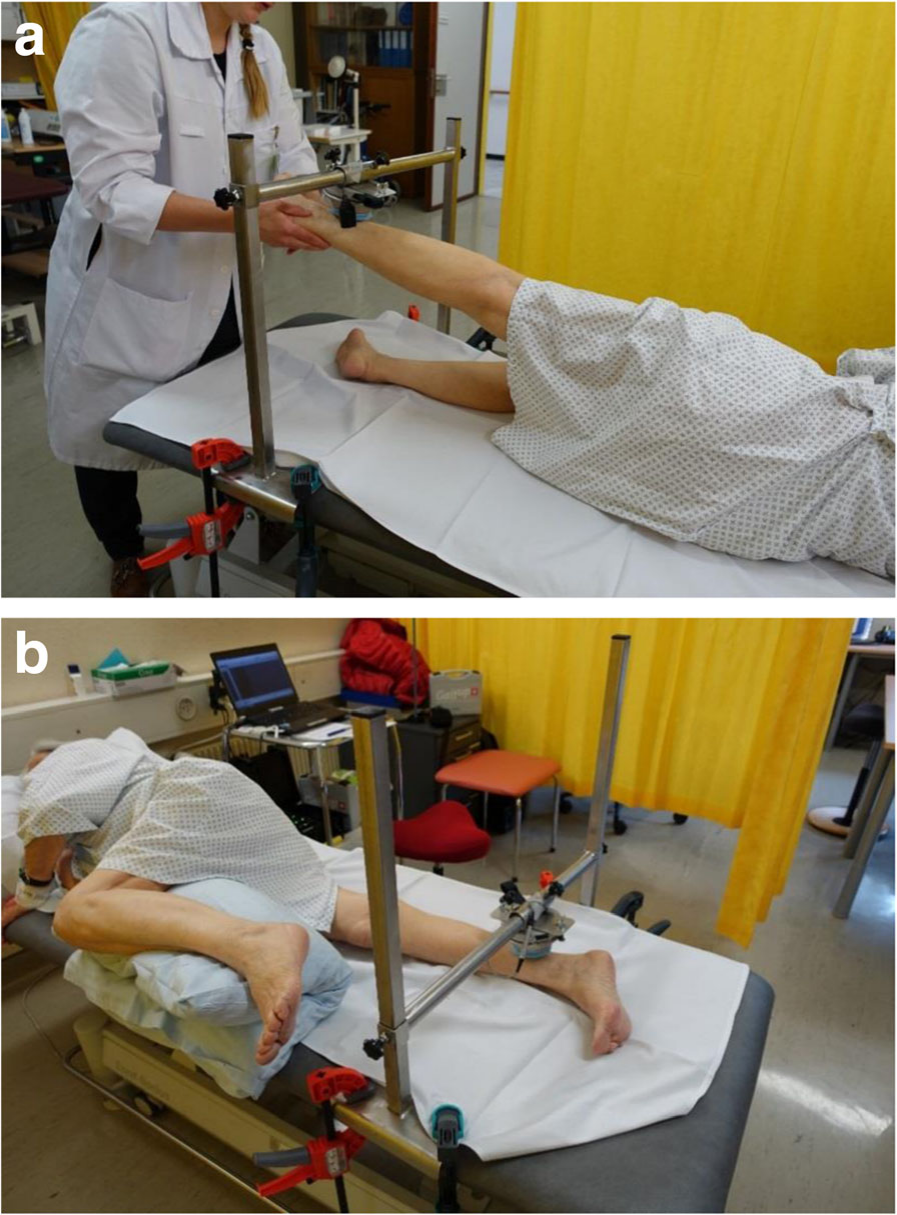
**a** For the hip abduction test the participant was in a side-lying position. The dynamometer was positioned in a 10° angled position to the horizontal line, five cm proximal the malleolus externus. **b** For hip adduction test the person was again in a side-lying position. This time the lower leg of the participant was tested. The starting position is at 0° adduction

For the hip abduction test the participant was in a side-lying position. The tester held the participant’s leg in a five to eight degree abduction position, close to the dynamometer which was attached to a frame fixed on the bed. The dynamometer was positioned in a 10° angled position to the horizontal line, five cm proximal the malleolus externus [31]. The participant was instructed to push his leg as quickly and forcefully as possible towards the dynamometer, to hold it with his maximum force for three seconds and to relax (Fig. 1a). Verbal encouragement was given during all measurements.

### Hip Adduction Test

For hip adduction testing, the person was again in a side-lying position. This time the lower leg of the participant was tested. The starting position was at 0° adduction and the participant was asked to push the leg in five degree adduction against the dynamometer fixed to a frame (Fig. 1b).

The participant had to repeat each test three times with a break of one minute between every trial. Verbal encouragement was given during all measurements.

### Data processing

**Fig. 2:**
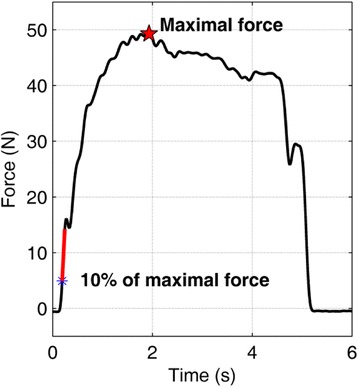
The rate of force change was evaluated over 50 ms after 10% of the MVIS was reached

The raw force signals were exported to Matlab (Mathworks, Natick, MA), which was used for data analysis and statistics. The signal was low-pass filtered (75 ms moving average) to attenuate high-frequency noise. The MVIS was defined as the peak value reached within zero to four seconds. The rate of force change was evaluated over 50 ms after 10% of the MVIS was reached (see Fig. 2 for graphical explanations). Both MVIS and RFG were normalized by body mass [32, 33].

### Statistics

We used descriptive statistics to summarize the characteristics of the study population. The number of participants who could successfully finish the hip abductor and adductor MVIS and RFG test are expressed as a percentage and the time to administer the test is expressed in mean and SD. Scatter plots were used to display the individual results of both testing sessions. The identity line (test = retest) is shown to get a better estimation of the dispersion of the results and of potential bias between sessions.

ICC_agreements_ were used to assess the repeatability of each dependent variable (MVIS, RFG) between both measurement sessions. ICC_agreement_ represents the proportion of between-subject variance as compared to total variance. Three ICC’s_agreement_ were computed in Abduction and Adduction groups: one from the whole sample (total), one for the subgroup of fallers, and one for the subgroup of non-fallers. The averages of the six trials within each session (three trials with the left leg and three trials with the right leg) were used as two within-subject repetitions. The ICC method was that proposed by McGraw and Wong [34]. We applied the ICC(A,1) model, which assesses the degree of agreement among measurements assuming a two-way random effects model. In addition, we computed 95% confidence intervals (CI) through bootstrapping (5000 resamples, bias corrected and accelerated percentile method).

In addition the group level estimation of the within-subject average variability [35], i.e., the standard error of measurements (SEM), was computed as follows: $S E M={S}_T\sqrt{1}- I C C$, where *S*_*T*_ is the standard deviation of the whole sample including both sessions. The smallest detectable difference (SDD) was also computed: $S D D= S E M\cdot 1.96\cdot \sqrt{2}$, it is the smallest change that could be considered significant. The SDD was normalized by the mean and expressed as percentage.

## Results

Overall 76 older subjects were recruited. Half of them (19 fallers and 19 non-fallers) underwent the abduction and the other half (19 fallers and 19 non-fallers) the adduction test. The characteristics of the whole study population as well as the characteristics per subgroup are presented in Table 1.

**Table 1:** Description in mean (SD) of the whole population and the two subgroups recruited for the hip abduction and hip adduction strength test

Study Population	All (*N* = 76)	Non-fallers (*N* = 38)	Fallers (*N* = 38)
Sex: F/M	41/35	22/16	19/19
Age (years)	80.46 (7.05)	78.47 (6.64)	82.45 (6.98)
Weight (kg)	67.58 (12.58)	69.25 (10.68)	65.92 (14.18)
BMI	24.89 (3.95)	25.00 (3.04)	24.78 (4.73)
SPPB^a^	8.45 (3.07)	10.45 (2.37)	6.41 (2.27)
Subgroup for abduction test	Non-fallers (*N* = 19)	Fallers (*N* = 19)	*P*-value
Sex: F/M	11/8	9/10	
Age (years)	79.84 (7.08)	81.37 (7.67)	0.528
Weight (kg)	68.15 (12.01)	66.17 (15.15)	0.658
BMI	24.71 (3.39)	23.84 (4.40)	0.500
SPPB	10.00 (2.54)	6.37 (2.03)	<0.001
ABD MVIS (N/kg)	1.23 (0.49)	0.77 (0.37)	<0.01
ABD RFG (N/kg/s)	7.65 (5.27)	3.498 (2.51)	<0.01
Subgroup for abdduction test	Non-Fallers (*N* = 19)	Fallers (*N* = 19)	*P*-value
Sex: F/M	11/8	10/9	
Age (years)	77.11 (6.04)	83.53 (6.23)	<0.01
Weight (kg)	70.35 (9.36)	65.66 (13.55)	0.222
BMI	25.28 (2.72)	25.72 (4.99)	0.743
SPPB^a^	10.89 (2.16)	6.44 (2.55)	<0.001
ADD MVIS (N/kg)	1.40 (0.43)	0.93 (0.51)	<0.01
ADD RFG (N/kg/s)	5.61 (2.99)	2.80 (2.46)	<0.01

*Abd* Abduction, *Add* Adduction, *MVIS* Maximum Voluntary Isometric Strength, *RFG* Rate of Force Generation, *N* Newton, *kg* Kilogram, *s* Seconds, *SPPB* Short Physical Performance Battery

^a^One faller of the adduction group didn’t realize this test

All recruited persons could successfully complete the tests. To measure one muscle group (MVIS and RFG hip abduction or adduction of both sides (left and right)) a mean time of 10.58 ± 1.56 min was necessary.

**Fig. 3:**
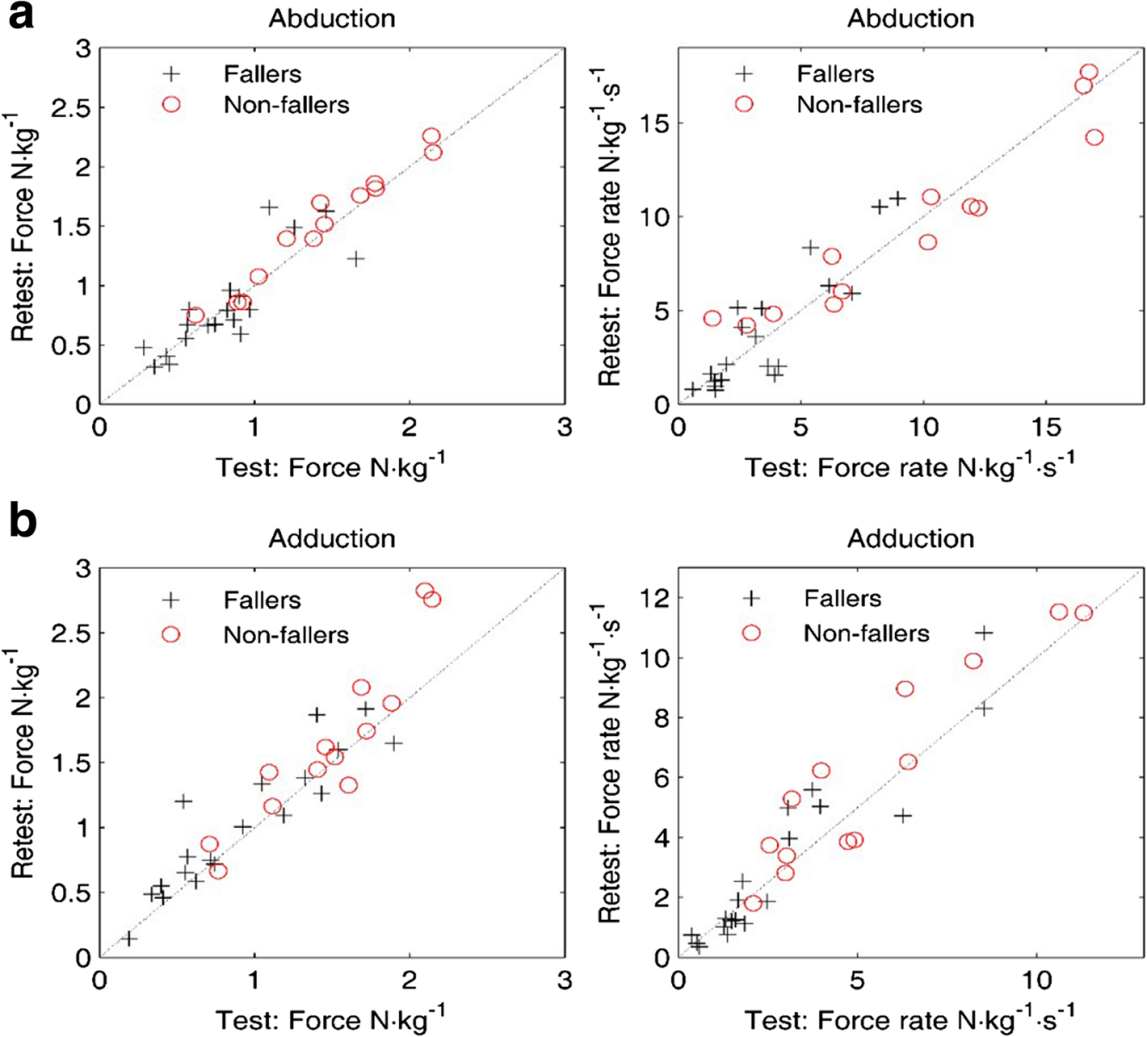
**a** and **b**: Scatterplot indicating the relationship between the first and second test of **a**) hip abduction strength respectively **b**) adduction strength for fallers and non-fallers

The reliability of the hip abduction and adduction test is represented in Table 2 and Fig. 3. The ICC’s_agreement_ for hip abduction were 0.84 [0.72–0.94] for fallers and 0.97 [0.94–0.99] for non-fallers for the MVIS and 0.85 [0.70–0.93] for fallers and 0.96 [0.92–0.98] for non-fallers for the RFG measures. The ICC’s_agreement_ for hip adduction were 0.85 [0.70–0.93] for fallers and 0.96 [0.92–0.98] for non-fallers for the MVIS and 0.83 [0.72–0.94] for fallers and 0.91 [0.76–0.97] for non-fallers for the RFG measures.

**Table 2:** Intraclass correlation coefficients (ICC’s_agreement_), Standard error of measurement (SEM) and smallest detectable change (SDD) of the hip abductor and hip adductor strength test

	ICC [95%CI]	SEM [unit of measure]	SDD [%]	ICC [95%CI]	SEM [unit of measure]	SDD [%]	ICC [95%CI]	SEM [unit of measure]	SDD [%]
	All (*N* = 38)			Fallers (*N* = 19)			Non-Fallers (*N* = 19)		
ABD									
MVIS	0.94 [0.87–0.97]	0.12	32%	0.84 [0.72–0.94]	0.15	51%	0.97 [0.94–0.99]	0.08	18%
RFG	0.94 [0.91–0.97]	1.05	51%	0.85 [0.70–0.93]	1.12	81%	0.96 [0.92–0.98]	1.01	37%
ADD									
MVIS	0.90 [0.84–0.95]	0.17	39%	0.90 [0.72–0.96]	0.16	44%	0.87 [0.79–0.94]	0.19	35%
RFG	0.94 [0.90–0.97]	0.77	48%	0.93 [0.84–0.97]	0.72	67%	0.93 [0.84–0.98]	0.83	39%

*ABD* Abduction *ADD* Adduction, *MVIS* Maximum Voluntary Isometric Strength (N/kg), *RFG* Rate of Force Generation (N/kg/s), *CI* Confidence Interval

The SEM and SDD for hip abduction MVIS measures of the total sample was 0.12 N/kg, respectively 32.6% and 1.05 N/kg/s, respectively 51.7% for hip abduction RFG measures. The SEM and SDD for hip adduction MVIS measures of the total sample was 0.17 N/kg, respectively 39.6% and 0.77 N/kg/s, respectively 48.9% for hip adduction RFG measures.

Subgroup analyses showed a SEM for hip abduction MVIS measures of 0.15 N/kg for fallers and 0.08 N/kg for non-fallers and SEM for hip abduction RFG measures of 1.12 N/kg/s for fallers and 1.01 N/kg/s for non-fallers. The SDD of hip abduction MVIS measures was 51.8% for fallers and 18.1% for non-fallers. The SDD of hip abduction RFG measures was 81.8% for fallers and 37.2% for non-fallers.

For the subgroup who performed the hip adduction test the SEM of MVIS measures was 0.16 N/kg for fallers and of 0.19 N/kg for non-fallers; respectively of 0.72 N/kg/s for fallers and of 0.83 N/kg/s for non-fallers for RFG measures. The SDD was 44.9% for fallers and 39.8% for non-fallers for MVIS measures and 67.7%, respectively 45.9% for RFG measures.

The scatterplot (Fig. 3a and b) shows the individual results of both testing sessions and gives an estimation of the dispersion of the results. We observed that more points are above the identity line than below.

## Discussion

The results of this study show that the measure of hip abductor and adductor strength in elderly people is practicable with a comparable good reliability in fallers and non-fallers.

The ICC’s_agreement_ can be explained by a high inter-individual variability in hip abduction and adduction strength in our population which minimizes the influence of the intra individual variability in the population and hence, allows to recommend this measurement approach for research in a comparable population.

The set-up that we used to measure hip abductor and adductor strength was the same as the set-up used by Widler et al. and Nadler et al. [28, 29]. Widler et al. [29] tested the reliability of unilateral hip abductor strength assessments in sixteen healthy young subjects in three different body positions with the use of a stabilized commercial dynamometer in the side-lying, supine, and standing positions. The highest MVIS value for each side and position was retained and strength data provided by the dynamometer were consistently normalized to body weight. The retest took place 48 to 72 h after the first assessment and it was assumed that the participants’ characteristics did not change in that time interval. The maximal hip abductor strength was significantly higher in the side-lying position compared with the standing and supine positions. The test-retest reliability of strength measurements in terms of ICC_agreement_ in their young population was of 0.90 in the side-lying position which is very similar to the ICC_agreement_ that we calculated in our population (ICC_agreement_ = 0.94). However, 95% confidence intervals were not presented which would allow a full comparison with our population. Nadler et al. [28] also assessed the reliability of hip abductor strength in ten subjects aged from 25 to 35 years. In their study an inexperienced examiner did three consecutive measures. The procedure was repeated two weeks later by the same subjects with an evaluator blinded to the initial results. For each of the hip muscles tested, an average and maximal value was computed and presented from the three test repetitions obtained. The ICC’s_agreement_ in their study were again similar to the results that we had in older adults and ranged from 0.95 to 0.98. Again, these authors did not present a 95% confidence interval of their ICC’s_agreement_ which would allow a comparison of the reliability obtained for their young and our older adults. In our study we computed the mean value of three left and three right trials before calculating the ICC’s_agreement_. Additionally, we also calculated the ICC’s_agreement_ based on the trial with the best performance and compared it with the ICC’s_agreement_ calculated based on the average. No relevant differences were found between these two approaches. Widler et al. and Nadler et al. [28, 29] did not assess the reliability of the hip abduction RFG and the hip adduction MVIS and RFG, therefore, we could not compare our values with theirs. Nevertheless, reliability outcomes of hip adductor MVIS and reliability coefficients of RFG measures in our study were similar to reliability outcomes of the hip abduction MVIS. In addition, Widler et al. and Nadler et al. [28, 29] did not present the SDD or the SEM which could give an indication if the test can be used in daily practice.

The high inter-subject variability for hip abduction strength measures in our population and the relatively high inter-subject variability in hip abduction strength measures in healthy young subjects confirm that hip abductor strength is a variable with clinical relevance. Nadler et al. [28] calculated a coefficient of variation for hip abduction strength of 26% in young subjects whereas the inter-subject variability in our study with older persons was of 49%.

The SEM, i.e., the intra-subject variability, was high in our study. For the overall group we had a SEM of 0.12 N/kg, respectively 1.05 N/kg/s for the hip abduction and of 0.19 N/kg, respectively 0.77 N/kg/s for the hip adduction. The SDD for hip abduction in the overall group was 32.6% for the MVIS and 51.6% for the RFG. For adduction the values were 43.2 and 56.1% respectively. The variability that we also observed between the three measures of one single test session (results not shown) confirms that the performance of older adults varies a lot. In addition, we could observe a small (not significant) learning effect between the first and second test series which might have contributed to the moderate to large SEM and SDD.

Even if the ICC’s_agreement_ were high, physical therapists should be careful when using this measure for assessing the progress of an individual person in a daily clinical use due to the high SEMs and SDDs [35]. The progress of an individual person in hip abduction strength must be above 32% for the MVIS and above 39% for the RFG to be considered as real. Still, the clinical relevance of changes greater than the SDD remain to be evaluated.

One solution could be to combine several tests to increase reliability. Through the Spearmann-Brown prophecy formula, we can predict that averaging two separated measurement sessions would improve SDD for adduction from 39 to 29% for the MVIS and from 48 to 35% for the RFG [36].

The strength of this study is that we used a test procedure which is feasible and which can be tolerated by older persons. No specific training is necessary. Compared to the isokinetic strength assessment tools, our procedure is less time-consuming and more practical for a geriatric population, and equally reliable to isokinetic strength assessments in young soccer player [37]. In addition we provide all necessary statistics to get a clear overview of the reliability of the test procedure.

The following limitations should be noted. We only assessed the intra - tester reliability and performed the two tests on the same day. The assessor was not blinded to the subgrouping (faller/non-faller). The fact that we observed more points above the identity line than below for hip adduction might indicate a small learning effect. In addition, the current set-up and the high SEM and SDD do not indicate that the test is easy to use in a daily setting. Even if the time needed to test a muscle group is short (approximately 10 min), this remains slightly too long for daily clinical use. Due to this we should rethink the testing position, as the installation of the person in a side-lying position takes at least half of the time needed to perform the whole test. In addition it would be pertinent to assess how much a patient can progress in his MVIS and RFG in order to make sure that our measure can detect this change. It might be that more practice trials would decrease the SEM and SDD and thus improve the sensitivity to measure a change.

Future studies should be done to investigate different elements of reliability in a larger sample to repeat our findings. In addition, validity studies on different aspects should be done. Validity studies should particularly focus on discriminant validity in order to assess if hip frontal strength measures are able to discriminate between elderly fallers and non-fallers. The differences observed in this study between these subgroups would suggest that hip frontal plane strength is of clinical relevance. If this can be confirmed such a test would enable clinicians to gather crucial information about a directly targetable and trainable parameter related to fall risk. In addition researchers should investigate the ability of hip abductor and adductor strength measures to assess changes over time after a specific strength training program (treatment effect). We are aware that discrimination and evaluation of change are two totally different abilities and that it is difficult for a measure to combine both features in an optimal way. Nevertheless, both abilities are relevant in the context of fall prevention and should therefore not be neglected.

## Conclusion

In conclusion we can say that measures of hip abductor and adductor strength in older persons are feasible and reliable. We encourage physical therapists to routinely assess hip frontal plane strength as it provides an interesting new goal for clinical practice which could lead to the development of more specific treatment protocols for this population. However, the significance of moderate changes in these measurements may be limited by the large SDD and SEM. Therefore, physical therapists should be careful when using this measure for assessing the progress of an individual person in a daily clinical.

## Abbreviations

ABD: Abduction
ADD: Adduction
DSP: Distal Symmetric Neuropathy
ICC: Intraclass Correlation Coefficient
MVIS: Maximum Voluntary Isometric Strength
RFG: Rate of Force Generation
SDD: Smallest Detectable Difference
SEM: Standard Error of Measurement
SPPB: Short Physical Performance
UST: Uni-pedal Stance Time
